# A Study of Standardizing Frequencies Using Channel Raster for Underwater Wireless Acoustic Sensor Networks

**DOI:** 10.3390/s21165669

**Published:** 2021-08-23

**Authors:** Changho Yun, Suhan Choi

**Affiliations:** 1 Korea Research Institute of Ships & Ocean Engineering (KRISO), Daejeon 34103, Korea; sgn0178@kriso.re.kr; 2 Department of Mobile Systems Engineering, Dankook University, Yong-In 16890, Korea

**Keywords:** acoustic frequency band, bandwidth, channel raster, Internet of Underwater Things (IoUT), standardization, underwater cognitive acoustic networks (UACNs), underwater wireless acoustic sensor networks (UWASNs)

## Abstract

In this paper, we propose the method to standardize acoustic frequencies for underwater wireless acoustic sensor networks (UWASNs) by applying the channel raster used in the terrestrial mobile communications. The standardization process includes: (1) Setting the available acoustic frequency band where a channel raster is employed via the frequency specification analysis of the state-of-the art underwater acoustic communication modems. (2) Defining the center frequencies and the channel numbers as a function of channel raster, and the upper limit of the value of channel raster. (3) Determining the value of the channel raster suitable for the available acoustic frequency band via simulations. To set the value, three performance metrics are considered: the collision rate, the idle spectrum rate, and the receiver computational complexity. The simulation results show that the collision rate and the idle spectrum rate according to the value of channel raster have a trade-off relationship, but the influence of channel raster on the two performance metrics is insignificant. However, the receiver computational complexity is enhanced remarkably as the value of channel raster increases. Therefore, setting the value of channel raster close to its upper limit is the most adequate in respect of mitigating the occurrence of a collision and enhancing the reception performance. The standardized frequencies based on channel raster can guarantee the frequency compatibility required for the emerging technologies like the Internet of Underwater Things (IoUT) or the underwater cognitive radio, but also improves the network performance by avoiding the arbitrary use of frequencies.

## 1. Introduction

In the terrestrial mobile communications such as Universal Mobile Telecommunications System (UMTS), Long-Term Evolution (LTE), and 5G, signals are transmitted only at predefined frequencies, rather than at arbitrarily selected frequencies. In those systems, the transmit frequency is determined among one of the standardized frequencies. These selected frequencies can be expressed as a function of channel raster, a basic unit of frequency interval of a frequency grid which is used to transmit signals for wireless systems.

The size of a channel raster differs according to the type of mobile communications, and respectively specified in the European Telecommunications Standards Institute (ETSI) technical specifications [1,2,3]. The use of standardized frequencies based on channel raster can guarantee frequency compatibility among communication systems by allowing to use only pre-determined frequencies, which results in avoiding or mitigating interferences among the systems. Furthermore, the speed of frequency synchronization can be faster, and frequency resources can be utilized more efficiently.

On the other hand, the acoustic frequencies for underwater wireless acoustic sensor networks (UWASNs) have been freely used; all vendors and developers of acoustic communication systems have used desired acoustic frequencies without restrictions. This is due to the limited usage of underwater acoustic communications compared to the vast underwater region and the resulting non-competitiveness among nodes.

Currently, a variety of applications are being developed for UWASNs, which include scientific observation, the exploitation of ocean resources, disaster detection, military surveillance, leisure activities, and subsea construction monitoring [4,5,6]. The increase and diversification of underwater applications can intensify the demand for underwater acoustic communications and thus boost the competition for frequency use among nodes [7,8]. This situation can cause poor acoustic frequency utilization, as well as performance deterioration due to severe interference because all nodes can become the sources of interferences with each other.

Up to now, a plethora of network protocols have been proposed to enhance the overall performance of UWASNs, which involve medium access control (MAC), routing, localization, time synchronization, initialization, power control, and network management protocols as outlined in [9,10,11]. In particular, MAC protocols have been prevalently developed to resolve interference and competition among nodes in a harsh underwater communication environment [12,13,14,15]. With the help of MAC protocols, interference avoidance and collision-free transmission among nodes can be achievable so that a narrow acoustic frequency band can be efficiently utilized and overall network throughput can also be improved [16,17]. However, the MAC protocols developed so far are only focused on the exclusion of interference and contention among nodes connected to a system with the same transmission and reception frequencies. The use of overlapped frequency can still be unavoidable among nodes in different communication systems using different frequencies, which can cause interference and collision among them. Consequently, it is necessary for UWASNs to employ standardized frequencies to prevent severe competition and interference induced by unplanned and arbitrary use of acoustic frequencies.

Moreover, the emerging technologies such as the Internet of Underwater Things (IoUT) or the underwater cognitive acoustic networks (UCANs) requiring the frequency compatibility are developed for UWASNs. The IoUT provides users with various applications by interconnecting several systems equipped with different acoustic communication modules [18,19]. Thus, the use of unified frequencies is a strong requirement to accommodate heterogeneous systems in the IoUT platform. In the UCANs, a global frequency reference is also necessary to detect efficiently whether any portion of the spectrum is occupied or not, and to adjust their frequencies intelligently to use the idle frequencies without causing interferences [20]. Therefore, the frequency compatibility is crucially important to realize those new technologies for UWASNs.

Accordingly, we study how to support the frequency compatibility for UWASNs where the usage policy of acoustic frequencies is absent. To do this, we apply the channel raster concept of the terrestrial mobile communications to UWASNs because the center frequencies and the channel numbers for communications can be easily and clearly defined provided that channel raster is determined.

Before determining a channel raster for UWASNs, we need to primarily set the adequate acoustic frequency band where the channel raster is applied. The frequency band of underwater acoustic communications ranges normally from hundreds of Hz to hundreds of kHz, which is remarkably lower than that of terrestrial counterparts [21]. Furthermore, the entire acoustic frequency band can hardly be used, but it is mainly divided into three sub-bands: the low frequency band (<1 kHz), the middle frequency band (several kHz to several tens of kHz), and the high frequency band (~hundreds of kHz) according to the type of applications [22,23,24]. This implies that it can be inefficient to apply a specific channel raster equally to all sub-bands.

For example, if a channel raster considering the low frequency band only is equivalently employed to all sub-bands, the network performance may be deteriorated because the frequency grid induced by the channel raster is too dense in the middle frequency band or even the high frequency band. Therefore, in this study, we analyze the frequency specifications of commercial and developed acoustic communication modems to determine the most densely populated frequency band as the target frequency band where the channel raster is applied. This frequency band is also denoted as “available acoustic frequency band” throughout this paper.

After determining the available acoustic frequency band, the channel numbers and the center frequencies existing within the band are individually defined as a function of channel raster in order to form a frequency grid as specified Chapter 5 in [2].

Finally, the value of a channel raster for the available acoustic frequency band is heuristically determined through simulations because, as far as we know, there are no methods and criteria to set up the value in the literatures. We consider the number of concurrent transmitting nodes and their system bandwidths as main simulation parameters, which affect the performance such as the collision rate and the idle spectrum rate. By changing these parameters, the performances of the two cases where channel raster is used and not used are compared to investigate the feasibility of the use of channel raster. Then, we finally suggest the value of the channel raster suitable for the available acoustic frequency band based on the simulation results.

If a standardized frequency is applied on the basis of channel raster for UWASNs, we can expect several advantages as follows.

- It guarantees the frequency compatibility among heterogeneous systems by supporting a global frequency reference.
- When signals are transmitted and received at the standardized frequencies, the network throughput can be enhanced by preventing the prevalent occurrence of a collision caused by occupying frequencies arbitrarily.
- Underwater acoustic communications require high energy efficiency due to limited battery life and high replacement cost. When channel raster is applied, the process of frequency synchronization can be simplified and the resources for corresponding processing can be also reduced. Thus, overall energy efficiency can be improved.
- In the UCANs, cognitive users (or secondary users) can detect whether any portion of the spectrum is occupied or not efficiently and adjust their frequencies with much less complexity.

We organize the rest of this paper as follows. In Section 2, we determine the available acoustic frequency band where channel raster is employed, and define the channel numbers and the center frequencies as a function of channel raster. In Section 3, we verify the feasibility of channel raster for UWASNs and set its value via simulations. Section 4 concludes this paper.

## 2. Determining Available Acoustic Frequency Band and Defining Center Frequencies and Channel Numbers

In this section, we determine the available acoustic frequency band, where channel raster is applied by investigating the acoustic frequencies on which most underwater acoustic communication modems are operated. Then, we define the center frequencies and the channel numbers as a function of channel raster as in [2].

### 2.1. Determining Available Acoustic Frequency Band

To the best of our knowledge, Ref. [25] is the most recent work that analyzed the specifications of the state-of-the-art commercial and developed acoustic communication modems. In [25], the specifications of acoustic communication modems developed up to 2020 are summarized. The specifications include center frequency, system bandwidth, communication distance, data rate, modulation method, operating depth, transmission and reception powers, weight, temperature, and bit error rate. To determine the available acoustic frequency bandwidth, we focus on the center frequency and the system bandwidth specifications. Thus, we consider the acoustic modems of which frequency specifications are clearly specified in the analysis.

**Figure 1:**
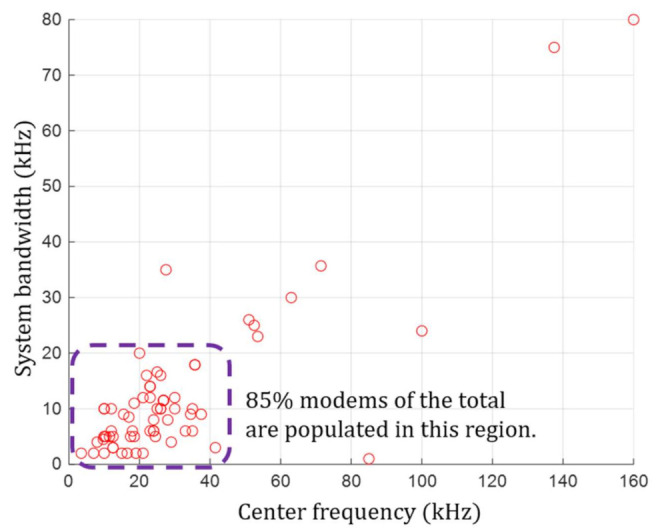
The scatter plot illustrating the pairs of center frequency and system bandwidth of underwater acoustic communication modems specified in [25].

Figure 1 is the scatter plot of the center frequency-system bandwidth pairs of underwater acoustic communication modems with the x-axis as the center frequency and the y-axis as the system bandwidth. It shows that 86.57% of modems have their center frequencies of 50 kHz or less. We also find that the same number of modems set their system bandwidths to be 20 kHz or less. The modems satisfying both the center frequency of 50 kHz or less and the system bandwidth of 20 kHz or less account for 85% of the total. As a result of this analysis, it can be seen that the majority of the modems determine their center frequency and system bandwidth within a specific range. Moreover, there is no modem of which center frequency is below 1 kHz. Based on the analysis result, we determine the available acoustic frequency band as [1 kHz, 50 kHz] with the bandwidth of 49 kHz and use it for our study. In addition, the system bandwidth should be less than 20 kHz, as illustrated in Figure 1.

### 2.2. Defining Center Frequencies and Channel Numbers Using Channel Raster

In this section, we create a frequency grid within the available acoustic frequency band by applying the concept of channel raster. As shown in Table 1, we describe all parameters for a frequency grid applied in the available acoustic frequency band.

**Figure 2:**
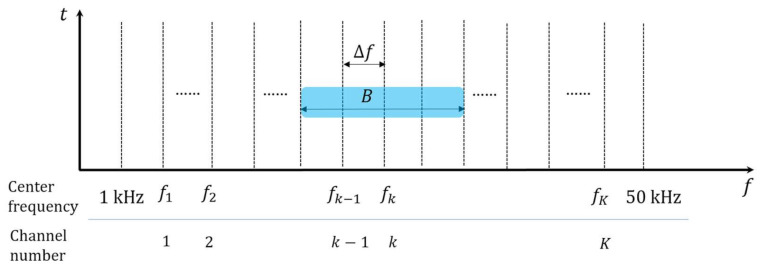
The illustration of the frequency grid in the available acoustic frequency band.

Figure 2 illustrates the frequency grid in the available acoustic frequency band. As obtained in Section 2.1, the available acoustic frequency band is from 1 kHz to 50 kHz, and thus the length of the band is 49 kHz. All of center frequencies for communications must be selected on the frequency grid and cannot be set beyond this band, and the number of channels is determined according to the value of channel raster.

By using Δf, the number of channels in the available acoustic frequency band can be derived as $K=\lfloor \frac{49\;\mathrm{kHz}}{\Delta f}\rfloor$ where ⌊x⌋ denotes a floor function, i.e., the greatest integer less than or equal to x. This implies that there are K channels in the available acoustic frequency band (i.e., 1≤k≤K). The center frequency corresponding to the channel k is represented as $f_{k}=1000+k\times \Delta f\;\left[\mathrm{Hz}\right]$, and thus all center frequencies in the available acoustic frequency band form a frequency grid, as depicted in Figure 2. For example, if Δf=100 Hz, the number of channels is 490, i.e., K=490 and the center frequency of channel k is given by $f_{k}=1000+100\times \Delta f\;\left[\mathrm{Hz}\right]$.

As shown in the example of the system bandwidth in Figure 2, a system bandwidth should be set symmetrically around a specific center frequency. Thus, the boundary of the system bandwidth is also designed to be placed in the frequency grid. To do this, the value of a system bandwidth is set as a multiple of 2Δf. Using this relation, the minimum system bandwidth is expressed as $B_{\mathrm{MIN}}=2\Delta f$. In addition, as the value of channel raster cannot exceed the minimum system bandwidth, its upper limit can be obtained as $\Delta f\leq \frac{B_{\mathrm{MIN}}}{2}$ in consideration of $B_{\mathrm{MIN}}=2\Delta f$.

## 3. Setting the Value of Channel Raster via Simulations

In this section, we determine the value of channel raster for the available acoustic frequency band through simulations. For this purpose, we define the performance metrics and describe simulation conditions. Then, we investigate the feasibility of the use of channel raster for UWASNs and finally determine the value of channel raster based on the simulation results.

### 3.1. Performce Metrics

To determine the value of channel raster, we consider three performance metrics: (1) the collision rate (CR), (2) the idle spectrum rate (ISR), and (3) the receiver computational complexity (RCC).

#### 3.1.1. Collision Rate

**Figure 3:**
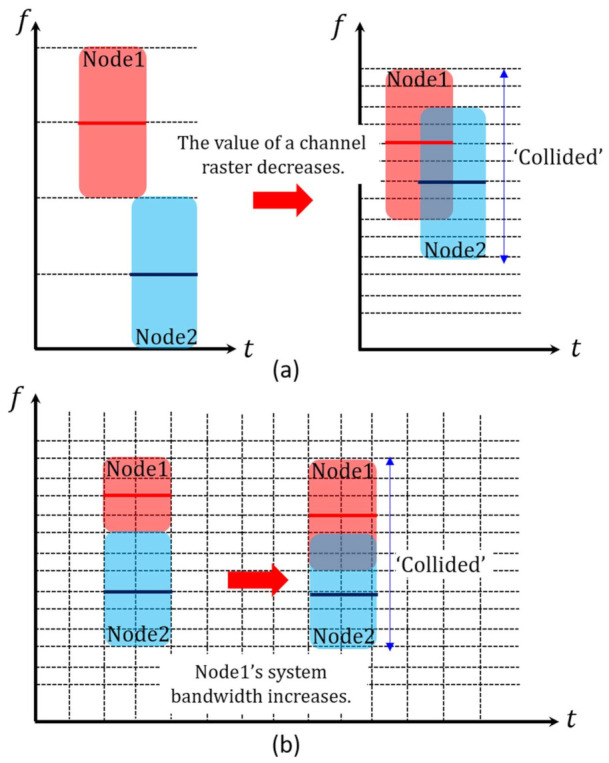
The illustration of the occurrence of a collision. (**a**) A collision induced by the decrease of the value of channel raster. (**b**) A collision induced by the increase of a node’s system bandwidth.

The collision rate is directly related to network throughput. That is, the lower the collision rate is, the higher network throughput can be guaranteed [26]. As illustrated in Figure 3a,b, any transmitting node can experience a collision if another node transmits its signal at the overlapped frequency band and time duration. Thus, we define the collision rate as the average rate that a node experiences a collision in the transmission mode during the entire simulation time. The average collision rate of a node can be expressed as

(1) $$CR=\frac{1}{N}\sum_{i=1}^{N}\frac{n_{Ci}}{n_{\mathrm{TX}i}},$$

where i, N, $n_{\mathrm{TX}i}$, and $n_{Ci}$ are the index of a node, the number of nodes, the accumulated number of transmissions of node i, and the accumulated number of collisions of node i during the simulation time, respectively.

The collision rate is affected by several factors such as the value of channel raster, system bandwidth, and the number of concurrent transmitting nodes. As shown in Figure 3a, if the value of channel raster decreases and thus center frequencies are densely distributed, the occurrence of a collision is more probable. As illustrated in Figure 3b, the increase of system bandwidth also boosts the collision probability. Finally, the increasing number of nodes transmitting simultaneously results in a severe collision. After all, the collision rate is the result of the combination of those influencing factors.

#### 3.1.2. Idle Spectrum Rate

The idle spectrum rate is an indicator to check the portion of idle frequency band in the available acoustic frequency band [27]. Normally, if specific frequency band is not occupied by any node, it is regarded as “idle”. Note that we also consider the frequency band “idle” in terms of frequency usage, if a collision occurs in that band and it is not used for successful communications (see Figure 3). Consequently, the idle spectrum rate can be expressed as

(2) $$ISR=\frac{1}{T}\sum_{t=1}^{T}\frac{IB_{t}}{49\;\mathrm{kHz}}$$

where t, T, and $IB_{t}$ are the discrete simulation time index, the length of simulations, and the amount of idle frequency band at t, respectively. $IB_{t}$ is obtained by using the usage results of all center frequencies in the available acoustic frequency band at each simulation time t.

The idle spectrum rate is also affected by the factors specified in Section 3.1.1, but it is unlikely to increase or decrease monotonically. For example, if the value of channel raster decreases, it is intuitively expected that the spectrum is likely to be used more efficiently. Accordingly, this can decrease the idle spectrum rate. However, the probability of the occurrence of a collision can also increase since center frequencies are densely located, increasing the idle spectrum rate. Therefore, this implies that there is a trade-off between the occurrence of a collision and the efficient usage of acoustic frequencies in view of the idle spectrum rate for a fixed value of channel raster.

In addition, the system bandwidth and the number of concurrent transmitting nodes have the similar effect on the idle spectrum rate. More specifically, as the system bandwidth and the number of transmitting nodes increase, the probability of a collision is expected to increase, resulting in the higher idle spectrum rate. However, if the system bandwidth and the number of transmitting nodes increase, the portion of unused frequency band can decrease, leading to the lower idle spectrum rate. Thus, it is hard to predict whether the idle spectrum rate can increase or decrease with respect to those factors.

#### 3.1.3. Receiver Computational Complexity

In UWASNs, the reliability of communications is challenging because of the inter-symbol interference (ISI) which could span tens or even hundreds of symbol durations [28]. Therefore, several communication techniques have been proposed, including single carrier with time-domain decision feedback equalizer (SC-TDE), orthogonal frequency-division multiplexing (OFDM), and single-carrier with frequency domain equalization (SC-FDE) [29]. The SC-FDE, in particular, is a promising low-complexity approach to mitigate the ISI by coping with long delay spread channels with single-tap equalizers. Besides, the SC-FDE has lower computational complexity than the SC-TDE [30]. In addition, although the computational complexity of SC-FDE is similar to that of OFDM, but the SC-FDE can avoid high peak-to-average power ratio (PAPR) and sensitivity to carrier frequency offset occurred in OFDM [30]. Therefore, the SC-FDE has been proposed for high-rate underwater acoustic communications [31,32,33,34].

The SC-FDE receiver consists of a down-converter, a fast Fourier transform (FFT) module, a channel estimator, a frequency domain equalizer, and an inverse FFT (IFFT) module, as illustrated in Figure 2 of [29]. Especially, we focus on the FFT process where the intensity of digitized signals is obtained in the frequency domain. The size of FFT has influence on the overall computational complexity of the FFT process; the larger the size of FFT, the higher the computational complexity. Accordingly, if the size of FFT increases, the overall receiver computational complexity also increases.

The size of FFT, denoted by n, is defined as $n=\frac{f_{s}}{\Delta F}$, where $f_{s}$ and ΔF are the sampling frequency and the frequency resolution, respectively [35]. The frequency resolution is an indicator of how densely the frequency components are analyzed in the FFT process. The sampling frequency should be at least twice the maximum detectable frequency (i.e., $f_{s}\geq 2f_{\mathrm{MAX}}$). The equation of the FFT size (i.e., $n=\frac{f_{s}}{\Delta F}$) shows that the size of FFT, which affects the receiver computational complexity, is inversely proportional to the frequency resolution. We note that if the relationship between frequency resolution and channel raster is defined, that between the receiver computational complexity and the channel raster can be also derived.

If a signal is transmitted only at the frequency grid based on channel raster, a receiver needs to detect the signals strength not at all frequencies but at the predefined frequencies only. Then, we can also check the signal intensity corresponding to the predefined frequencies in the FFT process provided that the value of channel raster (i.e., Δf) is higher than that of frequency resolution (i.e., Δf≥ΔF). In other words, we can set the frequency resolution of the FFT process based on the value of channel raster. Using this relation, the receiver computational complexity can be derived as a function of channel raster.

By taking into account three equations (i.e., $\Delta f\geq \Delta F,\;f_{s}\geq 2f_{\mathrm{MAX}}$, and $\Delta F=\frac{f_{s}}{n}$), we can obtain the relation between channel raster and the size of FFT as $n\geq \frac{2f_{\mathrm{MAX}}}{\Delta f}$ where n is a multiple of two. Then, the minimum FFT size can be derived as $n=\lceil \frac{2f_{\mathrm{MAX}}}{\Delta f}\rceil$ where the remainder of $\frac{n}{2}$ should be zero, and ⌈x⌉ denotes a ceiling function, i.e., the least integer greater than or equal to x. In [36], the receiver computational complexity is defined as a function of the FFT size as $\frac{n}{2}\log_{2}\left(n\right)$. By inserting $=\lceil \frac{2f_{\mathrm{MAX}}}{\Delta f}\rceil$ into $\frac{n}{2}\log_{2}\left(n\right)$, the receiver computational complexity considering the minimum FFT size is finally derived as

(3) $$RCC=\lceil \frac{f_{\mathrm{MAX}}}{\Delta f}\rceil \log_{2}\left(\left\lceil \frac{2f_{\mathrm{MAX}}}{\Delta f}\right\rceil \right)$$

Using the receiver computational complexity, we can also predict other receiver performances such as the reception processing time or the energy efficiency. If the receiver computational complexity is large, corresponding processing time and energy consumption also increase.

### 3.2. Simulation Operations and Conditions

The simulation is built using MATLAB software and its operations are as follows.

- We consider a UWASN consisting of multiple nodes which can be connected with each other via single-hop transmission with the capability of adjusting the transmission and reception frequencies in the available acoustic frequency band.
- The simulation is performed by generating nodes’ transmission events at each discrete time (DT). A node is either in the transmission mode or in the reception mode which is randomly determined at each DT. The state of a node is maintained only during one DT.
- This simulation is mainly targeted for investigating the pure influence of channel raster, so that any network protocol such as medium access control (MAC), routing, or flow control is not considered. Instead, the state of a node is determined in a random manner between two modes (i.e., transmission mode and reception mode) at each DT.
- Once a node is in the transmission mode, it determines its center frequency and system bandwidth, as specified in Section 2.2.
- The channel state is assumed to be error-free. Thus, a node in the reception mode is capable of successfully decode the received signal unless a collision occurs.
- After executing transmissions at each DT, corresponding results are updated, including the success of transmission per transmitting node and the usage status of the available acoustic frequency band.
- The operation executed at one DT is repeated during the total simulation time, the target performance measures are finally obtained using the updated results.
- Simulations are also executed case by case with respect to simulation conditions such as the value of channel raster, the number of transmitting nodes, and the system bandwidth through the same simulation operation.

The simulation conditions are given as follows:

- The total simulation time (T) is $10^{6}$ which implies that the same simulation process is performed $10^{6}$ times.
- The number of nodes (N) is 50.
- The number of concurrent transmitting nodes ($N_{\mathrm{TX}}$) is set 5:5:50. That is, we consider ten values of $N_{\mathrm{TX}}$ by increasing the value by 5 from 5 to 50.
- The available acoustic frequency band is [1 kHz, 50 kHz] as defined in Section 2.1.
- The system bandwidth is randomly determined between the minimum system bandwidth ($B_{\mathrm{MIN}}$) and the maximum system bandwidth ($B_{\mathrm{MAX}}$). The value of $B_{\mathrm{MIN}}$ is set in consideration of the maximum value of $N_{\mathrm{TX}}$. More specifically, when the value of $N_{\mathrm{TX}}$ is maximum, the system bandwidth of each node is set as $B_{\mathrm{MIN}}$, and thus $B_{\mathrm{MIN}}$ is given as 1 kHz because all the transmitting nodes should share the available acoustic frequency band fairly without a collision. Also, we consider two $B_{\mathrm{MAX}}$ values below 20 kHz as 5 kHz and 10 kHz in order to check the performance change according to the system bandwidth.
- The value of channel raster, Δf, is set to be less than the minimum system bandwidth ($B_{\mathrm{MIN}}$) which is given as 1 kHz, and thus given as 50:50:$\frac{B_{\mathrm{MIN}}}{2}$ [Hz]. That is, we consider ten values of Δf by increasing the value by 50 from 50 to 500.

In addition, we compare two methods Type 1 and Type 2 to check the feasibility of applying channel raster; Type 1 is the method employing channel raster, and Type 2 is that without channel raster. The two methods are described as follows.

- Type 1: The center frequency is randomly determined among the predefined center frequencies of a frequency grid. The system bandwidth is also randomly set between the minimum system bandwidth and the maximum system bandwidth, but it should be a multiple of twice the channel raster.
- Type 2: The center frequency is randomly determined among all frequencies in the available acoustic frequency band, and the system bandwidth is also randomly set between the minimum system bandwidth and the maximum system bandwidth.

### 3.3. Simuation Results

#### 3.3.1. Collision Rate

First, the simulation results and the influence of the value of channel raster on the collision rate (CR) when the maximum bandwidth ($B_{\mathrm{MAX}}$) of 10 kHz are summarized as follows.

**Figure 4:**
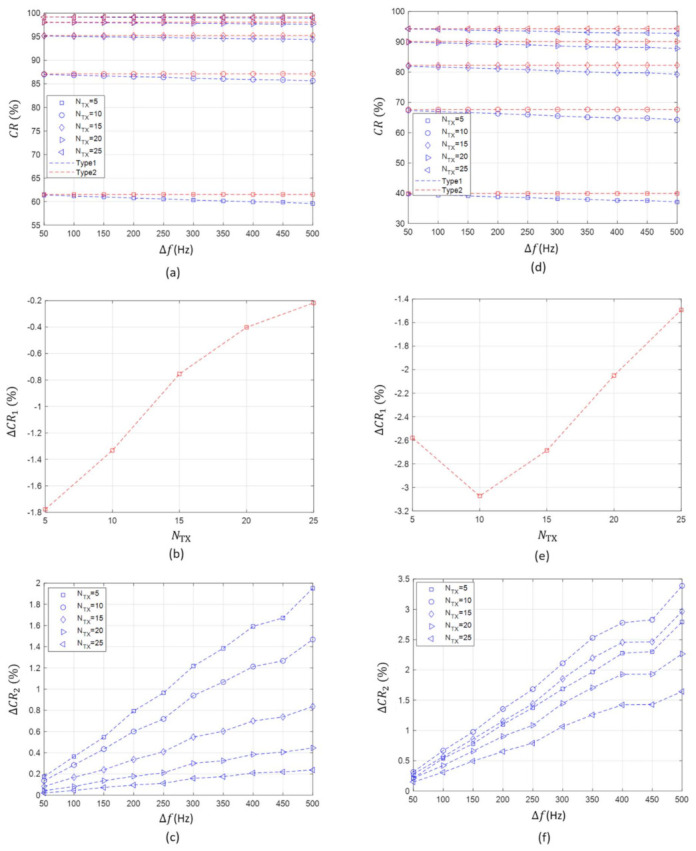
The *CR* of Type 1 and Type 2 according to Δf, $N_{\mathrm{TX}},\;\mathrm{and}\;B_{\mathrm{MAX}}$. (**a**) *CR* according to Δf, $N_{\mathrm{TX}}\;\mathrm{at}\;B_{\mathrm{MAX}}=10\;\mathrm{kHz}$. (**b**) $\Delta CR_{1}$ $\mathrm{at}\;B_{\mathrm{MAX}}=10\;\mathrm{kHz}$. (**c**) $\Delta CR_{2}\;\mathrm{at}\;B_{\mathrm{MAX}}=10\;\mathrm{kHz}$. (**d**) *CR* according to Δf, $N_{\mathrm{TX}}\;\mathrm{at}\;B_{\mathrm{MAX}}=5\;\mathrm{kHz}$. (**e**) $\Delta CR_{1}\;\mathrm{at}\;B_{\mathrm{MAX}}=5\;\mathrm{kHz}$. (**f**) $\Delta CR_{2}\;\mathrm{at}\;B_{\mathrm{MAX}}=5\;\mathrm{kHz}$.

- Before explaining the collision rate results, we describe all figures in Figure 4. Figure 4a illustrates the collision rate of Type 1 and Type 2 according to the value of channel raster (Δf) and the number of concurrent transmitting nodes ($N_{\mathrm{TX}}$) when the maximum system bandwidth ($B_{\mathrm{MAX}}$) is 10 kHz. Figure 4b shows the difference between the collision rate of Type 1 at Δf=500 Hz and that at Δf=50 Hz with respect to $N_{\mathrm{TX}}$, which is denoted by $\Delta CR_{1}$. Figure 4c displays the difference between the collision rate of Type 1 and that of Type 2 according to Δf and $N_{\mathrm{TX}}$, which is denoted by $\Delta CR_{2}$. Figure 4d–f correspond to Figure 4a–c, respectively, and show the collision rate results when $B_{\mathrm{MAX}}$ is 5 kHz.
- It was confirmed that the value of channel raster and the collision rate are inversely proportional as described in Section 3.1.1. This is due to the fact that the separation of the center frequencies in the available acoustic frequency band increases providing that the value of channel raster increases. This decreases the probability of a collision among the transmitting nodes.
- Figure 4a does not clearly show the effect of channel raster on the collision rate. Therefore, we compared the collision rate at the minimum value of channel raster (i.e., Δf=50 Hz) with that at the maximum value of channel raster (i.e., Δf=500 Hz), as shown Figure 4b. Under the given conditions, the absolute value of the difference in the collision rates between Δf=500 Hz and Δf=50 Hz ranges from 0.2% to 1.8% according to the number of transmitting nodes. The smaller the number of the transmitting node, the greater the influence of channel raster on the collision rate. This is because the collision rate approaches 1 regardless of the value of channel raster when the number of transmitting nodes increases. In other words, most of the transmitting nodes experience collisions, making the influence of channel raster become unremarkable.
- As a result, there is a slight difference in the collision rate of up to 2% even when the value of channel raster is changed. Thus, it can be concluded that the value of channel raster does not significantly affect the collision rate performance.

Second, the collision rate comparison between Type 1 and Type 2 in the case of $B_{\mathrm{MAX}}$ of 10 kHz is described as follows.

- It can be seen that the case using channel raster can reduce the occurrence of a collision slightly more than the case without channel raster, as illustrated in Figure 4c.
- Even when simulations are performed by changing the value of channel raster and the number of transmitting nodes, the collision rate difference between Type 1 and Type 2 is less than 2%, as illustrated in Figure 4c. This result shows that the use of channel raster has little effect to reduce the collision rate significantly.
- From the results, we can see that the number of transmitting nodes has a greater effect on the collision rate performance than the value of channel raster.

Third, the collision rate in the case of $B_{\mathrm{MAX}}$ of 5 kHz is also analyzed as follows.

- When the maximum system bandwidth decreases to 5 kHz, the effect of channel raster on the collision rate is still similar; as the value of channel raster increases, the collision rate decreases, as depicted in Figure 4c.
- However, simulation results show that the collision rate in the case of $B_{\mathrm{MAX}}$ of 5 kHz is lower than that in the case of $B_{MAX}$ of 10 kHz. We can find the difference between the two cases by comparing Figure 4a,d.
- The influence of channel raster on the collision rate becomes more remarkable. As illustrated in Figure 4e, the absolute value of the difference in the collision rates between Δf=500 Hz and Δf=50 Hz ranges from 1.4% to 3.2% according to the number of transmitting nodes.
- As depicted in Figure 4f, the collision rate difference between Type 1 and Type 2 also increases (i.e., 0.3~3.5%). Nevertheless, it shows a minor performance improvement of less than 4% even when a channel raster is applied.
- As shown in Figure 4a,d, the number of transmitting nodes and the maximum system bandwidth have a greater effect on the collision rate than the value of channel raster.

Overall, based on the simulation results it can be concluded that the influence of channel raster on the collision rate performance is insignificant under the given conditions.

#### 3.3.2. Idle Spectrum Rate

First, the idle spectrum rate performance in the case of $B_{\mathrm{MAX}}$ of 10 kHz is outlined as follows.

**Figure 5:**
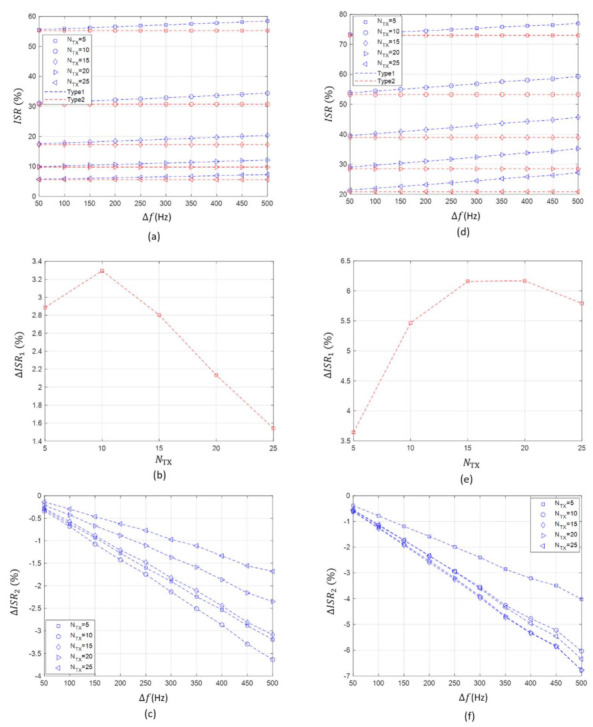
The *ISR* of Type 1 and Type 2 according to Δf, $N_{\mathrm{TX}},\;\mathrm{and}\;B_{\mathrm{MAX}}$. (**a**) *ISR* according to Δf, $N_{\mathrm{TX}}\;\mathrm{at}\;B_{\mathrm{MAX}}=10\;\mathrm{kHz}$. (**b**) $\Delta ISR_{1}\;\mathrm{at}\;B_{\mathrm{MAX}}=10\;\mathrm{kHz}$. (**c**) $\Delta ISR_{2}\;\mathrm{at}\;B_{\mathrm{MAX}}=10\;\mathrm{kHz}$. (**d**) *ISR* according to Δf, $N_{\mathrm{TX}}\;\mathrm{at}\;B_{\mathrm{MAX}}=5\;\mathrm{kHz}$. (**e**) $\Delta ISR_{1}\;\mathrm{at}\;B_{\mathrm{MAX}}=5\;\mathrm{kHz}$. (**f**) $\Delta ISR_{2}\;\mathrm{at}\;B_{\mathrm{MAX}}=5\;\mathrm{kHz}$.

- As the same as Figure 4, the description of all figures in Figure 5 is given as follows. Figure 5a shows the idle spectrum rate of Type 1 and Type 2 according to Δf and $N_{\mathrm{TX}}$ when $B_{\mathrm{MAX}}$ is 10 kHz. Figure 5b displays the difference between the idle spectrum rate of Type 1 at Δf=500 Hz and that at Δf=50 Hz with respect to $N_{\mathrm{TX}}$, which is denoted by $\Delta ISR_{1}$. Figure 5c illustrates the difference between the idle spectrum rate of Type 1 and that of Type 2 according to Δf and $N_{\mathrm{TX}}$, which is denoted by $\Delta ISR_{2}$. Figure 5d–f also correspond to Figure 5a–c, respectively, and show the idle spectrum rate results when $B_{\mathrm{MAX}}$ is 5 kHz.
- Unlike the collision rate, it is shown that the idle spectrum rate is proportional to the value of channel raster, as depicted in Figure 5a. This result is different from the expected trade-off between the occurrence of a collision and the usage of frequencies in Section 3.1.2. From this result, it can be seen that the coarse frequency grid has a greater effect on the idle spectrum rate performance than the occurrence of a collision as the value of channel raster increases. Accordingly, there is a trade-off between the collision rate and the idle spectrum rate in terms of determining the value of channel raster.
- Figure 5b shows the effect of the value of channel raster on the idle spectrum rate performance. The absolute value of the idle spectrum rate difference between Δf=500 Hz and Δf=50 Hz ranges from about 1.4% to 3.4% with respect to the number of transmitting nodes. Similar to the collision rate, the variation of the idle spectrum rate is also insignificant according to the value of channel raster. This implies that the value of channel raster also does not have a remarkable influence on this performance metric.

As shown in Figure 5c, the smaller the number of transmitting nodes, the more remarkable influence of channel raster on the performance. This result is due to the fact that the idleness of frequencies includes both “unused” state and “useless” state due to collisions as defined in Section 3.1.2. Therefore, if the impact of a collision is reduced, the idle spectrum rate by channel raster can be more clearly checked.

- As illustrated in Figure 5a,d, the idle spectrum rate for Type 2 is slightly lower than that for Type 1. From this result, it can be seen that the use of channel raster does not use the frequency band more efficiently, but rather sparsely at the cost of reducing the collision rate.
- Through Figure 5b,c, although the value of channel raster or the number of transmitting nodes is changed under the given conditions, the idle spectrum rate difference between Δf=500 Hz and Δf=50 Hz and that between Type 1 and Type 2 are all less than 4% at most. This result shows that the value channel raster does not have a significant effect on the idle spectrum rate as the same as the collision rate.

Second, the idle spectrum rate in the case of $B_{\mathrm{MAX}}$ of 5 kHz is also described as follows:

- When the maximum system bandwidth decreases to 5 kHz, the effect of channel raster on the idle spectrum rate is still similar; as the value of channel raster increases, the idle spectrum rate also increases, as illustrated in Figure 5d.
- However, all performance measures increase, which include the idle spectrum rate (i.e., 20~80%), and the absolute value of the idle spectrum rate difference between Δf=500 Hz and Δf=50 Hz (i.e., 3.5~6.5%), and the idle spectrum rate difference between Type 1 and Type 2 (i.e., 0.5~7%), as illustrated in Figure 5e,f. The simulation results show that the effect of channel raster on the idle spectrum rate becomes more significant as the maximum system bandwidth decreases.
- As shown in Figure 5a,d, like the collision rate performance, it is also confirmed that the number of transmitting nodes and the maximum system bandwidth have a greater effect on the idle spectrum rate than channel raster.

#### 3.3.3. Receiver Computational Complexity

While the collision rate and the idle spectrum rate are obtained through simulations, the receiver computational complexity performance was investigated numerically by using Equation (3) derived from Section 3.1.3. The results are described as follows.

**Figure 6:**
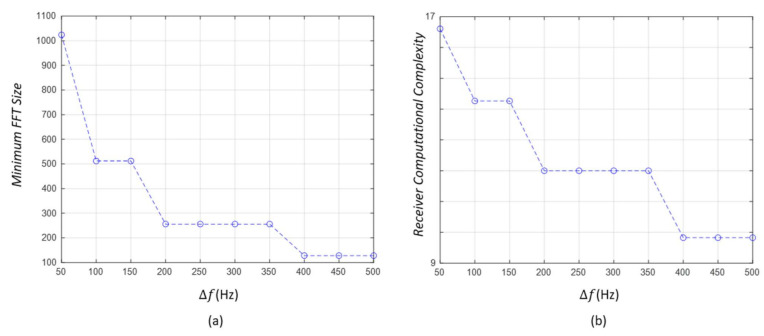
The receiver performance according to Δf. (**a**) Minimum FFT size. (**b**) RCC.

- Figure 6a,b illustrate the minimum FFT size and the receiver computational complexity (RCC) according to Δf, respectively.
- As illustrated in Figure 6a, the minimum FFT size decreases according to the value of channel raster. This is due to the fact that the larger the value of channel raster is, the coarser the frequency resolution can be set, which reduces the amount of data required for the FFT process.
- As the equation of the FFT size includes a ceiling function, the FFT size is a constant in a specific range of channel raster. Therefore, the FFT size decreases stepwise according to the value of channel raster, as shown in Figure 6a.
- It was also confirmed that the receiver computational complexity shows a similar performance pattern to the minimum FFT size with respect to the value of channel raster. That is, as the value of channel raster increases, the receiver computational complexity also decreases in a stepwise fashion.
- The receiver computational complexity also has a linear relationship with the energy consumption and processing time in the reception process. Therefore, it can be predicted that when the receiver computational complexity is high, both energy consumption and processing time also increases linearly.

#### 3.3.4. Setting the Value of Channel Raster and the Maximum System Bandwidth

Through the analysis of the collision rate, the idle spectrum rate, and the receiver computational complexity, we can summarize the simulation results as follows.

- There is a trade-off between the collision rate and the idle spectrum rate. The collision rate is improved as the value of channel raster increases, but the idle spectrum rate decreases conversely. However, the change of channel raster does not have a remarkable effect on the two performance metrics.
- In terms of the receiver computational complexity, which directly affects energy efficiency and processing time, the larger the value of channel raster is, the more significant the performance improvement is guaranteed.

Based on the simulation results, we propose that the value of channel raster should be set to improve the collision rate and the receiver computational complexity performances. Therefore, it is recommended that the value of channel raster should be set as close to its upper limit (i.e., $\frac{B_{\mathrm{MIN}}}{2}$) which is determined in Section 2.2.

On the other hand, the simulation results showed that the number of transmitting nodes and the system bandwidth have a greater effect on the collision rate and the idle spectrum rate than the value of channel raster. As we have no control over the number of transmitting nodes in general UWASNs, including unknown interferers, we can only adjust the maximum system bandwidth.

Determining the system bandwidth is also quite challenging, and thus can be another problem to solve for UWASNs. This is due to the fact that the system bandwidth needs to be set by considering various conditions such as channel status, network conditions, and network protocols. In this study, we show a simple example of setting the range of the maximum system bandwidth by considering only the collision induced by the overlapped use of spectrum.

In this regard, we determine the range of the maximum bandwidth which can guarantee the collision rate of less than 50% when no channel errors are taken into account. This is intended to find the conditions in which at least the half of the nodes can successfully use the desired frequency band even if multiple nodes set different system bandwidths and transmit signals at the same time.

To do this, the collision rate according to the number of transmitting nodes and the maximum system bandwidth is analyzed when the value of channel raster is 250 Hz. As it is confirmed that the value of channel raster does not have a significant effect on the collision rate, we analyze the performance by considering a specific channel raster value as 250 Hz.

**Figure 7:**
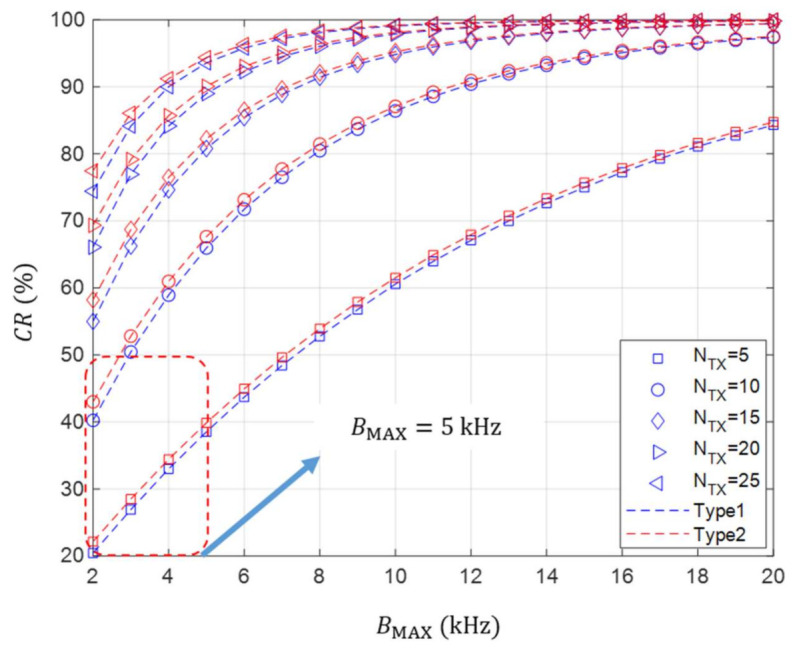
The collision rate according to the number of transmitting nodes and the maximum system bandwidth in case of Δf=250 Hz.

As illustrated in Figure 7, it is shown that it is infeasible to increase the maximum system bandwidth by more than 5 kHz when at least five nodes transmit signals simultaneously. This implies that it is difficult to guarantee the transmission success probability of 50% due to the occurrence of a collision. In [25], several acoustic communication modems with the system bandwidth of 20 kHz or higher are currently produced in the acoustic frequency band of 50 kHz or less. However, we suggest that the maximum system bandwidth should be limited to 5 kHz or less in order to keep the average collision rate at least 50% or less in a situation where the maximum number of transmitting nodes is difficult to predict.

In this study, we set the range of the maximum system bandwidth under the simplified scenario. However, determining the range of the maximum system bandwidth can be also another significant issue for UWASNs where extensive simulations need to be conducted in consideration of various target collision rates, channel errors or network protocols.

## 4. Conclusions and Discussions

The frequency band for underwater acoustic communications has been known as an “open spectrum” due to its scarcity of usage. However, the advent of diverse underwater applications and emerging technologies like the IoUT or the underwater cognitive radio are creating an interference problem induced by the use of acoustic frequencies without policies or regulations. This situation motivated us to create the standardized frequency system for underwater acoustic communications.

To standardize acoustic frequencies, we primarily set the available acoustic frequency band which ranges from 1 kHz to 50 kHz through the analysis of the frequency specifications of current commercial and developed underwater acoustic communication modems. Then, we defined the center frequencies and the channel numbers based on the concept of channel raster specified in terrestrial mobile communication systems standards such as LTE or 5G due to the lack of previous works corresponding to the standardization of underwater acoustic frequencies. Finally, we set the value of channel raster via simulations, and determined the frequency grid spaced apart by the channel raster in the available acoustic frequency band. We also proposed the adequate system bandwidth that allows more than the half of nodes to avoid collisions upon transmitting signals at the standardized center frequencies.

The use of standardized acoustic frequencies based on channel raster can guarantee the frequency compatibility required for the aforementioned emerging technologies, and enhance performances like the collision rate and the receiver computational complexity. Above all, the standardized frequency system can be used as a reference to make an unprecedented frequency channel planning for UWASNs.

Like terrestrial mobile communication systems in which a channel planning is designed according to frequency bands, the standardized frequency system was designed only targeted for the available acoustic frequency band, where the current underwater acoustic communication modems are prevalently used, in this study. As the demand for underwater acoustic communications is increasing in both low and high acoustic frequency bands, a research on determining the proper value of channel raster for these bands will be conducted in the ongoing study.

In addition, we can extend the standardization of acoustic frequencies in the aspect of the communication range. By nature of sound, low-frequency sound propagates farther than high-frequency sound. This implies that acoustic frequencies can be grouped according to the communication range such as low-communication range, mid-communication range, and high-communication range. Therefore, it is also necessary to determine the channel raster suitable for the frequency group corresponding to the communication range in our future work.

## Figures and Tables

**Table 1:** The description of parameters necessary to explain a frequency grid.

Parameters	Description
Δf	The value of channel raster
K	The maximum channel number
k	Channel number (1≤k≤K)
$f_{k}$	Center frequency corresponding to channel k
B	System bandwidth
$B_{\mathrm{MIN}}$	The minimum system bandwidth
$B_{\mathrm{MAX}}$	The maximum system bandwidth

## Data Availability

Not applicable.

## References

[1] Universal Mobile Telecommunications System (UMTS). UTRA Repeater Radio Transmission and Reception. https://www.etsi.org/deliver/etsi_ts/125100_125199/125106/10.02.00_60/ts_125106v100200p.pdf.

[2] LTE Evolved Universal Terrestrial Radio Access (E-UTRA). Base Station (BS) Radio Transmission and Reception. https://www.etsi.org/deliver/etsi_ts/136100_136199/136104/15.03.00_60/ts_136104v150300p.pdf.

[3] 5G. NR. Base Station (BS) Radio Transmission and Reception. https://www.etsi.org/deliver/etsi_ts/138100_138199/138104/15.02.00_60/ts_138104v150200p.pdf.

[4] Murad M., Sheikh A., Manzoor M., Felemban E., Qaisar S. (2015). A survey on current underwater acoustic sensor network application. Int. J. Comput. Theory Eng..

[5] Underwater Wireless Communication Market—Global Drivers, Opportunities, Trends, and Forecasts to 2022. https://www.globenewswire.com/news-release/2017/06/09/1011958/0/en/Underwater-Wireless-Communication-Market-Global-Drivers-Opportunities-Trends-and-Forecasts-to-2022.html.

[6] Jiang Z. (2008). Underwater acoustic network-Issues and solutions. Int. J. Intell. Control Syst..

[7] Wang Q., Dai H.N., Cheang C.F., Wang H. (2017). Link connectivity and coverage of underwater cognitive acoustic networks under spectrum Constraint. Sensors.

[8] Luo Y., Mo H., Zhu Y., Peng Z., Cui J.H. (2017). Receiver-initiated spectrum management for underwater cognitive acoustic network. IEEE Trans. Mob. Comput..

[9] Fattah S., Gani A., Ahmedy I., Idris M.Y.I., Hashem I.A.T. (2020). A survey on underwater wireless sensor networks: Requirements, taxonomy, recent Advances, and open research challenges. Sensors.

[10] Sharif-Yazd M., Khosravi M.R., Moghimi M.K. (2017). A survey on underwater acoustic sensor networks: Perspectives on protocol design for signaling, MAC and routing. J. Comput. Commun..

[11] Goyal N., Dave M., Verma A.K. (2019). Protocol stack of underwater wireless sensor network: Classical approaches and new trends. Wirel. Pers. Commun..

[12] Guqhaiman A.A., Akanbi O., Alijaedi A., Chow C.E. (2021). A survey on MAC protocol approaches for underwater wireless sensor networks. IEEE Sens..

[13] Li S., Qu W., Liu C., Qiu T., Zhao Z. (2019). Survey on high reliability wireless communication for underwater sensor networks. J. Netw. Comput. Appl..

[14] Garcia M., Sendra S., Atenas M., Lloret J. (2011). Underwater wireless ad-hoc networks: A survey. Mobile Ad Hoc Networks: Current Status and Future Trends.

[15] Afroz F., Braun R. (2020). Energy-efficient MAC protocols for wireless sensor networks: A survey. Int. J. Sens. Netw..

[16] Wei L., Guo Y., Cai S. (2018). MAC protocol for underwater acoustic sensor network based on belied state space. EURASIP J. Wirel. Commun. Netw..

[17] Jiang S. (2018). State-of-the-art medium access control (MAC) protocols for underwater acoustic networks: A survey based on a MAC reference model. IEEE Commun. Surv. Tutor..

[18] Domingo M. (2012). An overview of the internet of underwater things. J. Netw. Comput. Appl..

[19] Jahanbakht M., Xiang W., Hanzo L., Azghadi M.R. (2021). Internet of underwater things and big marine data analytics—A comprehensive survey. IEEE Commun. Surv. Tutor..

[20] Luo Y., Pu L., Zuba M., Peng Z., Cui J.H. (2014). Challenges and opportunities of underwater cognitive acoustic networks. IEEE Trans. Emerg. Top. Comput..

[21] Stojanovic M., Preisig J. (2009). Underwater acoustic communication channels: Propagation models and statistical characterization. IEEE Commun. Mag..

[22] Yun C., Choi S. (2021). A Study on channel bandwidth determination according to acoustic frequencies for underwater wireless cognitive networks. J. KICIS.

[23] Ahmad A.M., Kassem J., Barbeau M., Kranakis E., Porretta S., Garcia-Alfaro J. (2018). Doppler effect in the acoustic ultra-low frequency band for wireless underwater network. Mob. Netw. Appl..

[24] Kim S.M., Byun S.H., Kim S.G., Lim Y.K. Characterization of high-frequency underwater acoustic channel around 100 kHz in a shallow water. Proceedings of the IEEE Oceans.

[25] Zia M., Poncela J., Otero P. (2020). State-of-the-art underwater acoustic communication modems: Classifications, analyses and design challenges. Wirel. Pers. Commun..

[26] Alfouzan F.A. (2021). Energy-efficient collision avoidance MAC protocols for underwater sensor networks: Survey and challenges. J. Mar. Sci. Eng..

[27] Liu X., Lu W., Ye L., Li F., Zou D. (2017). Joint resource allocation of spectrum sensing and energy harvesting in an energy-harvesting-based cognitive sensor network. Sensors.

[28] Kilfoyle D.B., Baggeroer A.B. (2000). The state of the art in underwater acoustic telemetry. IEEE J. Ocean. Eng..

[29] He C., Xi R., Wang H., Jing L., Shi W., Zhang Q. (2017). Single Carrier with Frequency Domain Equalization for Synthetic Aperture Underwater Acoustic Communications. Sensors.

[30] Falconer D., Ariyavisitakul S.L., Benyamin-Seeyar A., Eidson B. (2002). Frequency domain equalization for single-carrier broadband wireless systems. IEEE Commun. Mag..

[31] Zheng Y.R., Xiao C., Yang T.C., Yang W.B. (2010). Frequency-domain channel estimation and equalization for shallow-water acoustic communications. Phys. Commun..

[32] He C., Huang J., Zhang Q., Shen X. Single carrier frequency domain equalizer for underwater wireless Communication. Proceedings of the the 2009 WRI International Conference on Communications and Mobile Computing.

[33] He C.B., Huang J.G., Zhang Q.F. Hybrid Time-Frequency Domain Equalization for Single-Carrier Underwater Acoustic Communications. Proceedings of the Conference on Under Water Networks WUWNet ’12.

[34] Xia M., Rouseff D., Ritcey J.A., Zou X. (2014). Underwater Acoustic Communication in a Highly Refractive Environment Using SC-FDE. IEEE J. Ocean. Eng..

[35] Semmlow J. (2017). Circuits, Signals and Systems for Bioengineers.

[36] Chen C., Wang Y., Liu X., Diao J. A power-efficient FFT preprocessing algorithm utilizing analysis filter bank. Proceedings of the SMIMA 2018.

